# Construction of a SNP Fingerprinting Database and Population Genetic Analysis of Cigar Tobacco Germplasm Resources in China

**DOI:** 10.3389/fpls.2021.618133

**Published:** 2021-02-24

**Authors:** Yanyan Wang, Hongkun Lv, Xiaohua Xiang, Aiguo Yang, Quanfu Feng, Peigang Dai, Yuan Li, Xun Jiang, Guoxiang Liu, Xingwei Zhang

**Affiliations:** ^1^Key Laboratory of Tobacco Improvement and Biotechnology, Tobacco Research Institute of Chinese Academy of Agricultural Sciences, Qingdao, China; ^2^Haikou Cigar Research Institute, Hainan Provincial Tobacco Company of China National Tobacco Corporation, Haikou, China

**Keywords:** cigar, single nucleotide polymorphism, KASPar assays, DNA fingerprinting, variety identification

## Abstract

Cigar tobacco is an important economic crop that is widely grown around the world. In recent years, varietal identification has become a frequent problem in germplasm preservation collections, which causes considerable inconvenience and uncertainty in the cataloging and preservation of cigar germplasm resources, in the selection of parental lines for breeding, and in the promotion and use of high quality varieties. Therefore, the use of DNA fingerprints to achieve rapid and accurate identification of varieties can play an important role in germplasm identification and property rights disputes. In this study, we used genotyping-by-sequencing (GBS) on 113 cigar tobacco accessions to develop SNP markers. After filtering, 580,942 high-quality SNPs were obtained. We used the 580,942 SNPs to perform principal component analysis (PCA), population structure analysis, and neighbor joining (NJ) cluster analysis on the 113 cigar tobacco accessions. The results showed that the accessions were not completely classified based on their geographical origins, and the genetic backgrounds of these cigar resources are complex and diverse. We further selected from these high-quality SNPs to obtained 163 SNP sites, 133 of which were successfully converted into KASP markers. Finally, 47 core KASP markers and 24 candidate core markers were developed. Using the core markers, we performed variety identification and fingerprinting in 216 cigar germplasm accessions. The results of SNP fingerprinting, 2D barcoding, and genetic analysis of cigar tobacco germplasm in this study provide a scientific basis for screening and identifying high-quality cigar tobacco germplasm, mining important genes, and broadening the basis of cigar tobacco genetics and subsequent breeding work at the molecular level.

## Introduction

Tobacco (*Nicotiana tabacum* L.), is an annual or limited perennial herbaceous plant species that belongs to the botanical family Solanaceae in the order Solanales. Tobacco is an important economic crop in many parts of the world, including China, and it is also one of the first model plants to be used in molecular biology and genetic engineering research. In China, cigar tobacco germplasm is very scarce, accounting for only 3% of tobacco germplasm resources, and most of the cigar varieties are imported from abroad. Because of the introduction channels for cigar tobacco germplasm in China, the varieties are diverse, and the different germplasm preservation institutions obtain cigar tobacco varieties from one another and name them independently. This has resulted in many problems such as repeated introductions of individual varieties, different varieties with the same name, varieties with many different names, and unclear genetic relationships among varieties. These problems have made it very difficult and inconvenient to catalog and preserve cigar tobacco germplasm resources, to select parental lines for breeding, and to promote excellent varieties for production. Over the last 30 years, the use of molecular markers has enabled the characterization and mapping of genes and metabolic pathways in plants, the study of species diversity and evolution, marker-assisted selection (MAS) in breeding, germplasm characterization, and estimation of seed purity (Dreisigacker et al., 2005; Jiang, 2015). Single-nucleotide polymorphisms (SNPs) have emerged as the most widely used genotyping markers due to their abundance in the genome and the relative ease in determining their frequency in a cost-effective manner in a large number of individuals (Varshney et al., 2009; Deschamps et al., 2012; Hayward et al., 2012).

Genotyping-by-sequencing (GBS) is a novel application of next-generation DNA sequencing for discovering SNPs that can be used for crop improvement (Poland and Rife, 2012; De Donato et al., 2013; Alipour et al., 2017). GBS is a simple, highly multiplexed system that can generate large numbers of informative SNPs for use in genetic analyses and genotyping (Beissinger et al., 2013). GBS is a reduced-representation genotyping strategy that is becoming increasingly important as a cost-effective tool for genomics-assisted breeding in a range of plant species (Narum et al., 2013; He et al., 2014; Lu et al., 2015; Uncu et al., 2016; Elbasyoni et al., 2018). GBS was originally developed for high-resolution association studies in maize and has been extended to a range of species with large and complex genomes. Unlike other high-density genotyping technologies which have mainly been applied to general interest “reference” genomes, the low cost of GBS makes it a powerful approach for discovering and genotyping SNPs in a variety of crop species and populations (Baldwin et al., 2012; Wu and Blair, 2017). GBS is a technically straightforward, highly multiplexed technology that is suitable for population studies, germplasm characterization, plant genetics, and breeding in diverse crops, and it has widely been applied to many crops with large genomes for which genomic resources are not well developed (Davey et al., 2011; Poland et al., 2012; Jaganathan et al., 2015).

SNPs have a high density across the entire genome, are genetically stable, and are also easy to adapt to automatic genotyping methods (Varshney et al., 2009; Paux et al., 2012; Gong et al., 2016). Many other high-throughput, low-cost SNP genotyping platforms have been developed, such as the GoldenGate (Fan et al., 2003) and Infinium platforms (Steemers and Gunderson, 2007), TaqMan by Life Technologies (Livak et al., 1995), and the KASPar system (KBiosciences Competitive Allele-Specific PCR). Many of these technologies have been widely used in important crop species such as wheat, maize, soybean, cowpea, and pea (Allen et al., 2011; Hiremath et al., 2012). Compared with other SNP genotyping methods, the KASPar system from KBiosciences has the advantages of high accuracy, strong site adaptability, and low cost, and it is suitable for detecting SNP sites in a large number of samples (Semagn et al., 2014). Along with the development of high-throughput DNA sequencing technology and bioinformatics data analysis, KASPar is well suited for large-scale automated detection and has a high application value in crop genetic diversity analysis and linkage map construction and can achieve full-process high-throughput detection typing. At present, KASPar has been applied in many species such as wheat (Trick et al., 2012), cotton (Byers et al., 2012), and rice (Yang et al., 2019).

DNA fingerprinting refers to the identification and detection of the different composition and lengths of variable genomic DNA sequences among varieties, which, like human fingerprints, are unique (Tian et al., 2015). DNA fingerprinting has the advantages of speed, accuracy, and precision and is thus a powerful tool for identifying varieties and strains (Gao et al., 2016). Over the past two decades, several different DNA marker technologies, including those based on restriction fragment length polymorphisms (RFLPs), amplified fragment length polymorphisms (AFLPs), inter-simple sequence repeats (ISSR), simple sequence repeat (SSR), and single-nucleotide polymorphisms (SNPs), have been widely used in research areas such as DNA genotyping of varieties, genetic diversity analyses, association studies, and molecular marker-assisted breeding (Powell et al., 1996; Nandakumar et al., 2004; Gunderson et al., 2005; Khampila et al., 2008; Semagn et al., 2012; Thomson et al., 2012; Sindhu et al., 2014). Of these markers, SNPs are one of the marker types recommended for constructing a DNA fingerprint database by the International Union for the Protection of New Varieties of Plants (UPOV) BMT Molecular Testing Guidelines (Button, 2008). Compared with traditional markers, SNP marker fingerprints have the characteristics of large number, wide distribution, high stability, ease of use, and rapid and high-throughput typing (McCouch et al., 2010; Primmer et al., 2010; Silva et al., 2012).

This study used GBS sequencing data from 113 cigar tobacco germplasm resources to develop SNP markers, and SNP sites with good polymorphism that were evenly distributed across the genome were identified and converted into KASP markers. The KASP system was used to genotype 96 cigar varieties (from 113 sequenced accessions), and the candidate markers were screened based on the genotyping results. Following this, the KASPar platform was used to genotype the candidate verification populations with 43 cigar tobacco varieties, and the genotypic data was obtained. Based on these genotyping results, the core markers and candidate core markers for fingerprinting cigar germplasm were screened. We then used the core markers to perform genotyping on the remaining cigar tobacco germplasm to obtain genotypic data. The core markers were used to construct DNA fingerprints of 216 cigar tobacco accessions. Following this, we performed population structure analysis and variety identification of the cigar tobacco germplasm collection.

## Materials and Methods

### Plant Materials

The experiment was carried out in the Genetic Breeding Laboratory of the Tobacco Research Institute of the Chinese Academy of Agricultural Sciences from December 2019 to July 2020. The GBS sequencing materials consisted of 113 cigar tobacco varieties (identified and registered) collected from major domestic breeding units (Supplementary Table 1). For core SNP marker verification, 43 cigar tobacco accessions collected from major domestic breeding units (Supplementary Table 2) and 216 cigar tobacco accessions were used for DNA fingerprinting (Supplementary Table 3). All materials were provided by the National Infrastructure for Crop Germplasm Resources (Tobacco, Qingdao) and Haikou Cigar Research Institute.

### DNA Extraction and GBS Library Construction

All 113 cigar accessions were subjected to reduced-representation genome sequencing using GBS. These 113 varieties represent germplasm from high-quality cigar-producing regions such as Cuba, Indonesia, the Dominican Republic, the United States, China, Brazil, Europe, and Southeast Asia. For each germplasm accession, seven vigorous plants were randomly selected at the seedling stage and young leaf samples totaling 0.5 g were collected in 2-ml centrifuge tubes, frozen immediately on dry ice, and transported to the laboratory where they were stored at −80°C until they were used for DNA extraction. The leaf samples were ground in a sample grinder, and genomic DNA was extracted using the RaPure Plant DNA Kit (Guangzhou Magen Biotechnology Co., Ltd.). Quality and concentration of DNA were determined using a NanoDrop2000 UV spectrophotometer (Thermo Scientific, MA, USA), and working solutions were prepared at a concentration of 30 ng/μl. DNA samples (100 ng/reaction) were double-digested with EcoRI and NIaIII (New England Biolabs, Ipswich, MA) in 96-well plates. The digested DNA samples were mixed with 25 pmol of the A1 and A2 adapters per well and ligated to add adapters to both DNA ends of the digested DNA fragments. Libraries were pooled, size-selected (400–600 bp) on a 1% agarose gel, column-purified using a PCR purification kit (NEB, Ipswich, MA, USA), and amplified for 12 cycles using Phusion DNA polymerase (NEB, Ipswich, MA, USA). Average fragment size was estimated on a Bioanalyzer 2100 (Agilent, Santa Clara, CA, USA) using a DNA1000 chip following a second column purification, and library quantification was performed using PicoGreen (Invitrogen, Carlsbad, CA, USA). The pooled libraries were adjusted to 10 nmol/μl, and 125 base paired-end reads (PE125) were generated using a Novaseq6000 sequencing instrument (Illumina, San Diego, CA, USA).

### SNP Identification and Development of KASP Primers

The BWA (Burrows–Wheeler Aligner) program was used to map the high-quality Illumina PE sequence reads to the tobacco reference genome (https://www.ncbi.nlm.nih.gov/genome/?term=common+tobacco%5Borgn%5D). The Unified Genotyper module of the software GATK (3.4–46) was used to perform variant detection of multiple samples on the processed comparison files. The detected variants were filtered using Variant Filtration. The filter parameters used were; -Window 4, -filter “QD <4.0 || FS> 60.0 || MQ <40.0,” -G_filter “GQ <20.” In addition, the filtered high-quality SNPs were used to annotate the SNP detection results with ANNOVAR software.

The obtained SNP loci were screened based on the following criteria: (1) they are evenly distributed on the 24 tobacco chromosomes; (2) no genotype data is missing; (3) the number of unmeasured materials at the site is <20 and the minor allele frequency (MAF) is ≥0.34; (4) the polymorphism information content (PIC) is >0.35; (5) the Hardy–Weinberg equilibrium (HWE)-tested *p*-value is ≥0.01; and (6) there are no other mutations 100 bp before and after the polymorphic site (Li et al., 2018). For each SNP site retained in the screening, the surrounding sequence was trimmed 100 bp before and after the SNP, and the KASP primers were developed and designed by LGC Genomics LLC (Beverly, MA, USA). For each KASP SNP, two allele-specific primers and one common primer were designed. Parameters for primer design were as follows: GC content <60%, melting temperature (Tm) between 55 and 62°C, and PCR product size no larger than 120 bp. There were only two choices of allele-specific primers immediately up- or downstream of the SNP site. Therefore, all primers were manually selected, and GC content and Tm were calculated using DNAstar v7.0. Primers carrying standard FAM- or VIC-compatible tails (FAM tail: 5′-GAAGGTGACCAAGTTCATGCT-3′; VIC tail: 5′-GAAGGTCGGAGTCAACGGATT-3′) with a targeted SNP at the 3′ end were synthesized by Invitrogen Trading (Shanghai). Primer mixes were prepared as recommended by Kbioscience: 46 μl ddH_2_O, 30 μl common primer (100 μM), and 12 μl of each tailed primer (100 μM). The total amplification reaction volume was 5 μl in a 384-well plate and consisted of 2.43 μl of V4 2 × KASP Master mix, 0.07 μl KASP primer mix, and 2.5 μl template (30 ng of genomic DNA) as described previously (Chao et al., 2009). PCR amplification was performed as follows: hot start at 94°C for 15 min, followed by 10 touchdown cycles (94°C for 20 s; touchdown starting at 61°C, −0.6°C per cycle, 60 s), then followed by 30 cycles of amplification (94°C for 20 s and 55°C for 60 s). Fluorescence detection of the reactions was performed using a BMG POLARstar Omega scanner, and the data were analyzed using KlusterCaller 3.4.1 software (KBioscience).

### Data Analysis

The genetic distance (*p*-distance) between the populations was calculated using the SNPs from each individual. The formula for calculating the p-distance between two individuals *i* and *j* is:

$$\begin{array}{l} D_{ij}=\frac{1}{L}\sum_{l=1}^{L}d_{ij} \end{array}$$

Where *L* is the length of the high-quality SNP region. Assuming that the allele at position 1 is A/C, if the genotypes of the two individuals are AA and AA, then *d*_*ij*_ = 0; if the genotypes of the two individuals are AA and AC or AC and AC, then *d*_*ij*_ = 0.5; if the two individual genotypes are AA and CC, then *d*_*ij*_ = 1. TreeBest software (http://treesoft.sourceforge.net/treebest.shtml) was used to calculate the distance matrix, and the neighbor-joining method was then used to construct a phylogenetic tree based on this distance matrix. Using the high-quality SNPs, the eigenvectors and eigenvalues were calculated using GCTA software (http://cnsgenomics.com/software/gcta/pca.html) based on the SNP differences between individuals, and the PCA distribution map was drawn using the R language (http://www.r-project.org/). Based on the SNP information obtained from the above analysis, the population structure was analyzed using PLINK (http://zzz.bwh.harvard.edu/plink/reference.shtml), and frappe software (http://med.stanford.edu/tanglab/software/frappe.html) was used to examine population genetic structure.

### SNP Selection and Generation of 2D Barcodes

Ninety-six cigar tobacco varieties were used to test the newly developed KASP primers, and those giving good genotyping results were selected as candidate markers; 43 varieties were used as candidate verification groups to verify the selected KASP primers; primers that gave better results were selected as the core primers, and the others were considered to be candidate core markers. The core and candidate core SNP primers were used to obtain genotypic data from the cigar tobacco accessions, and the online software Caoliaoerweima (http://cli.im/) was used to generate the 2D barcodes for 216 cigar varieties. The genotypes based on the SNP barcodes were entered, and the 2D barcodes were automatically generated. When the barcode is scanned, the genotype of each accession is shown.

## Results

### SNP Identification

The original raw Illumina reads obtained by GBS sequencing were subjected to quality control and data filtering to generate high-quality clean data as the basis of this study (Supplementary Table 4). On average, 96.48% of the reads had a base error rate of <1% (Q20), while 90.27% of the reads had a base error rate of <0.1% (Q30). The GC distribution was normal, and the sequencing quality was high. The 113 cigar tobacco accessions gave an average of 1,520 million high-quality sequences, and an average of 1,510 million sequences could be mapped to the tobacco reference genome. The average mapping ratio was 99.48%. The average comparison rate met the requirements of the sequence analysis, so the subsequent analyses could then be performed.

**Figure 1:**
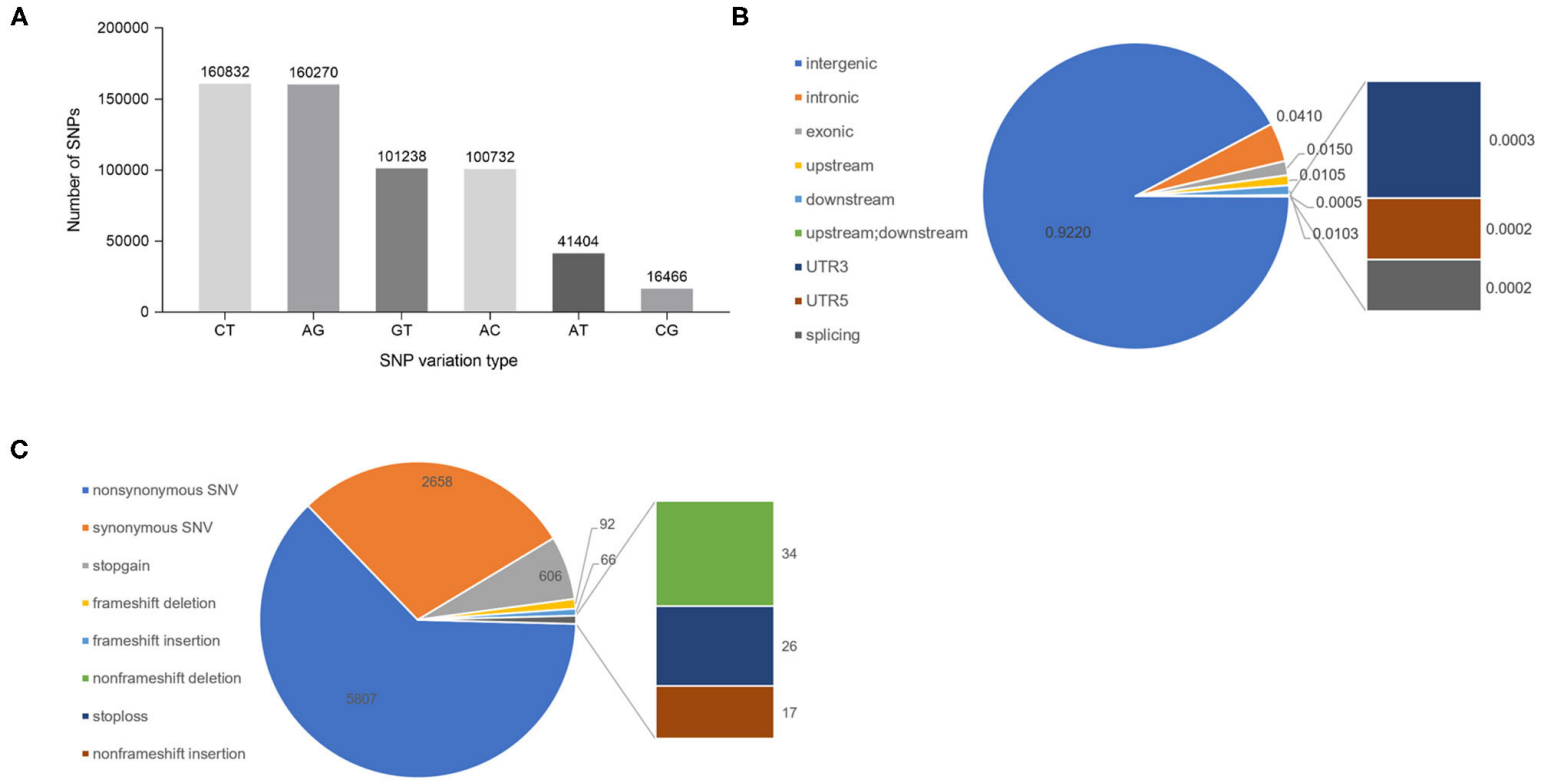
Analysis of SNPs in cigar tobacco genomes. **(A)** The six SNP types and the number of SNPs of each type. **(B)** The positions of the SNPs in the gene structures. Upstream: the SNP is located in the region 1 kb upstream (5′) of a gene; Downstream: the SNP is located in the 1-kb region downstream (3′) of a gene; splicing: variable splicing site within 2 bp. **(C)** Annotations of the SNPs in the exons. Non-synonymous SNV: a single-nucleotide change that causes an amino acid change; Synonymous SNV: a single-nucleotide change that does not cause an amino acid change; Stop gain: the mutation causes early termination of translation; Stop loss: the variation causes the loss of the terminator codon.

After completing the sequencing, SNP site variant identification on the tested germplasm was carried out using the GATK (Genome Analysis Toolkit; https://gatk.broadinstitute.org/hc/en-us) process; the identified SNPs were filtered, and 580,942 high-quality SNPs were obtained for subsequent analyses. Analysis of the types of predicted mutations of these SNPs showed that of the six possible single-base mutations, C/T and A/G transitions were the most frequent, accounting for 27.68% and 27.59% of the total, respectively. Of the 580,942 SNPs, 321,102 were transitions and 259,840 SNPs were transversions. The ratio of transitions to transversions was 1.24 (Figure 1A). Further analysis of the distribution of SNPs in the genome found that 92.20% were located in intergenic regions, 4.10% were located in introns, 1.50% were located in exons, 1.05% were located in the 1-kb region upstream of the transcriptional start site, 1.03% were located in the 1-kb region downstream of the transcription termination site, 0.05% were located in the 1-kb region upstream of one gene and at the same time located in the 1-kb region downstream of another gene, 0.03 and 0.02% were located in the 5′ and 3′ UTRs, and 0.02% were located in the splice junctions (Figure 1B). Functional annotation of the SNPs in the exonic regions of the genes is shown graphically in Figure 1C. There were 5,807 non-synonymous single-nucleotide variants (SNVs) that are predicted to cause changes in the encoded amino acid, and 2,658 synonymous SNVs that are predicted to not change the amino acid sequence. The ratio of non-synonymous SNVs to synonymous SNVs is 2.18. In addition, there are 606 SNPs that are predicted to lead to the early gain of a stop codon (stopgain) and 26 SNPs that lead to the loss of the stop codon (stoploss).

### Population Analysis

**Figure 2:**
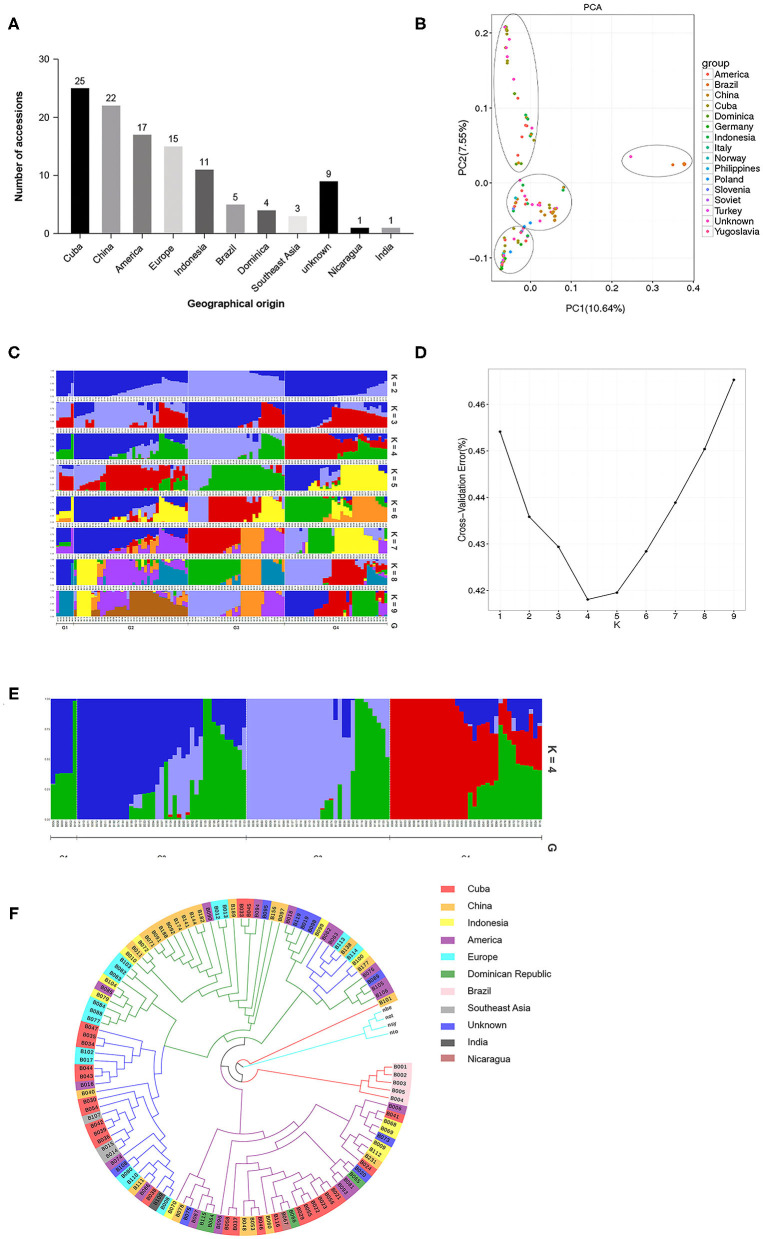
Population genetic analysis of 113 cigar tobacco accessions based on polymorphic SNP loci. **(A)** Geographical origins of the 113 cigar tobacco accessions used in this study. **(B)** Principal component analysis (PCA). **(C)** Population structure of the 113 cigar tobacco germplasm resources at different values of K. **(D)** Cross-validation error rates corresponding to different K values. **(E)** Population structure of the 113 cigar tobacco germplasm resources at *K* = 4. **(F)** An unrooted neighbor-joining (NJ) phylogenetic tree.

We used the R language package (http://www.r-project.org/) to perform PCA analysis using the sequences of the high-quality SNPs identified in the 113 cigar tobacco accessions. A two-dimensional graph based on the value of each sample in the first (PC1) and second (PC2) principal components is shown in Figure 2B. The values in brackets on the axis labels represent the percentage of the overall variation that is explained by PC1 and PC2, which was 10.64 and 7.55%, respectively. The 113 cigar accessions were divided into four well-separated clusters, and combined with the geographical origins of the varieties (Figure 2A), the PCA revealed that each of the four groups contained cigar tobacco accessions from different geographical locations. This shows that there is no significant correlation between the results of PCA and the geographical origin of the germplasm. We further used the Plink and frappe programs to analyze the population structure of the 113 cigar tobacco accessions. The number of clusters is usually determined based on the cross-validation error rate, and the number of clusters with the lowest cross-validation error rate is the optimal number of clusters. As shown in Figures 2C–E, the cross-validation error rate is the lowest when *K* = 4, which shows that the 113 cigar tobacco accessions can be divided into four clusters. Moreover, all the test materials were not completely classified according to their geographical origins, which is consistent with the phyletic classification results obtained from the PCA.

We also used the neighbor-joining method in treebest software to construct a phylogenetic tree for the 113 cigar tobacco accessions. As shown in Figure 2F, the four wild tobacco species included in the analysis (*Nicotiana benthamiana, Nicotiana otophora, Nicotiana sylvestris*, and *Nicotiana tomentosiformis*) cluster together and are clearly distinguishable from the 113 cigar cultivars in this study. The cigar tobacco accessions fall into four clades, and combined with the geographical data (Figure 2A), it can be seen that except for the variety B101 (WU MING Cigar), which is of unknown origin, and five cigar varieties from Brazil, they all cluster together. The remaining accessions do not fully cluster by geographical origin; the other three clades contain cigar tobacco accessions from different geographical regions, and the cigar tobacco accessions that originate from the same geographical region are also distributed across the three clades, but the clustering of cigar accessions from the same source within the clade indicates that the genetic backgrounds of these cigar tobacco accessions is complex and diverse. Cigar tobacco accessions from the same geographical region are both interrelated and independent.

### Design of KASP Primers and Selection of Core SNP Sites

**Figure 3:**
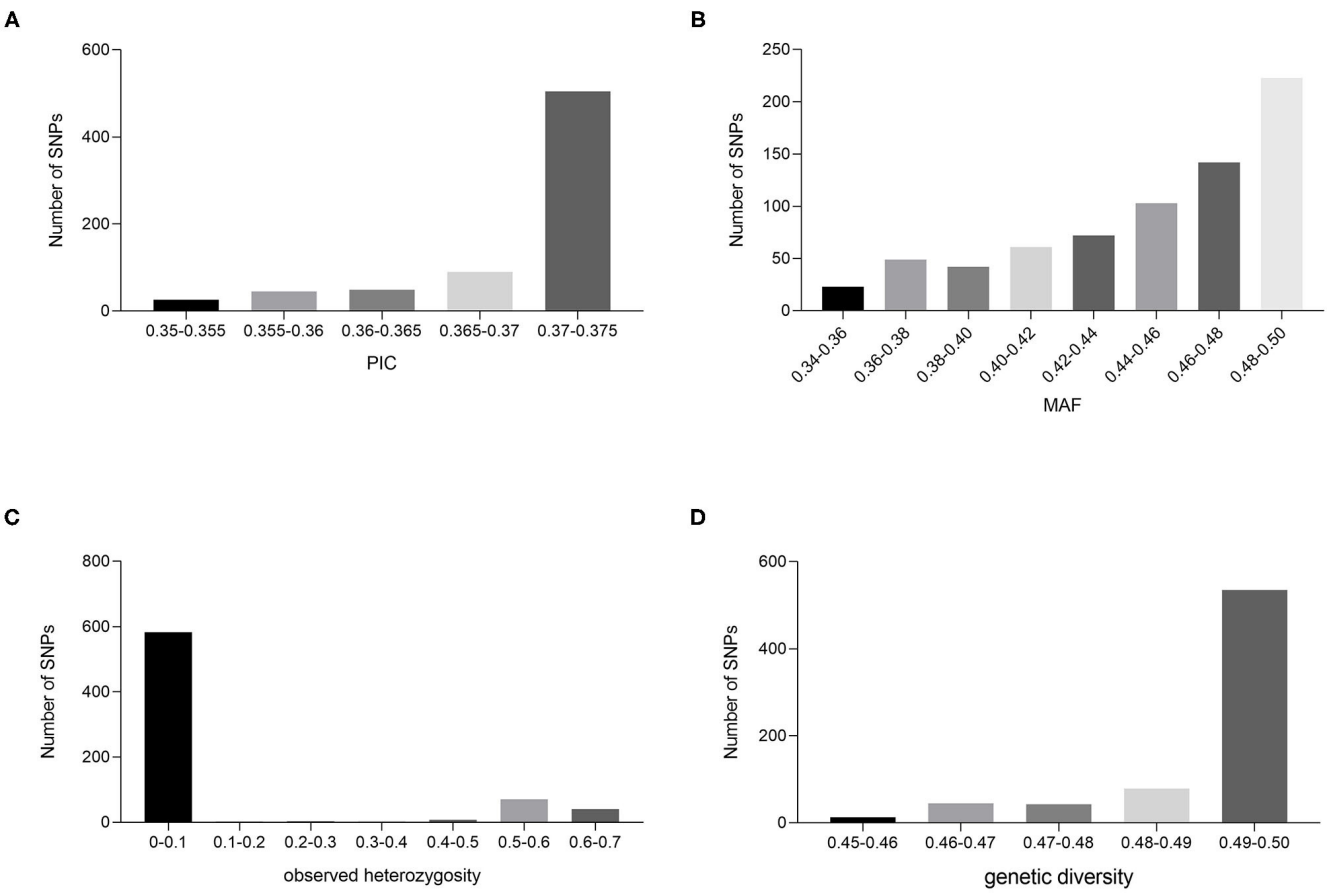
Population genetic analysis of cigar tobacco accession based on SNP loci. PIC **(A)**, MAF **(B)**, observed heterozygosity **(C)**, and genetic diversity **(D)** values for the 715 SNP markers based on data from 216 cigar tobacco accessions.

In the genome-wide SNP analysis, sites with indel and deletion rates >20% or MAF <5% were removed, leaving 108,267 sites. Sites with PIC <0.35 were removed, leaving 47,268 SNP sites. SNPs for which the Hardy–Weinberg equilibrium (HWE) tested *p*-value was <0.01 were removed, leaving 8,368 SNP sites. Plink 1.9 was used to remove one of the two loci in paired loci with high LD values by indep-Pairwise 50 10 0.2, leaving 2,982. Finally, SNP sites with other mutations 100 bp before and after the SNP were removed, leaving 737. The flanking sequences of these sites were extracted from the corresponding scaffold and matched to the reference genome at the chromosome level. Only the sites on the chromosomes were retained, resulting in 715 SNP sites for the next experiment. These 715 SNP sites showed good polymorphism and variety discrimination ability. The MAF and PIC values of the 715 SNP sites ranged between 0.346 and 0.500, and 0.350 and 0.375, and the average values were 0.45 and 0.37, respectively (Figures 3A,B). In particular, the percentage of PIC values between 0.370 and 0.375 was 70.6%, which suggests that these markers are strongly polymorphic. The observed heterozygosity for the 715 variable SNP sites ranged from 0 to 0.64, with an average of 0.10 (Figure 3C). The genetic diversity within the germplasm collection was also assessed and was found to range from a low of 0.45 to a high of 0.50, with an average of 0.49 (Figure 3D). The combined MAF, PIC, and observed heterozygosity values were used to further screen the SNP sites that are distributed across the 24 chromosomes, have no genotypic data missing, are mainly homozygous variant sites, and are detected in as many individuals as possible, which finally yielded 163 SNP sites. KASP primers were designed for all 163 SNP loci, and 133 (81.6%) were successfully converted into KASP markers.

The 133 KASP primer sets were typed and verified, and 96 cigar accessions from the 113 that were used for GBS were genotyped using the KASP system. Based on the typing results, we identified 76 KASP markers that showed high accuracy. Subsequently, 43 were used as candidate markers for screening of candidate verification populations, and the markers with better results were selected as core markers; 47 core KASP primer sets were screened (Supplementary Table 5), and 24 were designated as candidate core primers (Supplementary Table 6).

### Construction of DNA Fingerprints

**Figure 4:**
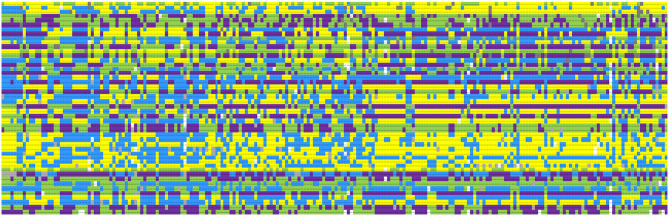
Fingerprints of 216 cigar tobacco accessions. Each line represents one SNP locus, and each column represents one accession. The SNP and cultivar information is given in Supplementary Tables 3, 5. Yellow, green, blue, and purple colors represent the nucleotides C/C, A/A, T/T, and G/G, respectively. Missing data is shown in gray. Heterozygous sites are shown in white.

The overall predictive accuracy of the 47-SNP barcode was 100% for the 216 cigar cultivars, and it can distinguish different materials (Figure 4). The online software Caoliaoerweima (http://cli.im/) was used to encode the genotypic data of the core SNPs for the 216 cigar tobacco accessions, and a 2D barcode fingerprint was constructed for each tobacco line (Supplementary File 1) that contains information such as variety name, type, botanical classification, and genotypic data. The fingerprints of these accessions were translated into a 2D barcode that is easily accessible using a cell phone. For example, B001 in Supplementary File 1 contains the following information: Name: Brazil-1; fingerprint code: C/C T/T T/T A/A A/A G/G T/T G/G T/T C/C G/G C/C A/A T/T G/G T/T G/G T/T T/T T/T G/G - T/T A/A C/C A/A C/C A/A A/A C/C C/C C/C C/C T/T C/C C/C C/C A/A - A/A G/T A/A T/T A/A C/C T/T A/A G/G.

### Identification of Cigar Germplasm Resources

Using the genotyping results for the 47 core SNP loci, NTSYS software was used to calculate a genetic distance matrix for the 216 tobacco accessions (Supplementary Table 7). The results showed that the genetic distance between cigar tobacco accessions ranged from 0 to 0.857. Among these accessions, the genetic distance between a group of three accessions (“TANG PENG,” “JIA MA SHAI YAN,” and “TIE CHI YAN”) and “LONG JIANG 851” was the highest, at 0.857. There were 41 markers that were polymorphic between them, indicating that they were the most genetically distant. The genetic distances between the following cultivar groups were all 0: “Brazil No. 1,” “Brazil No. 2,” “Brazil No. 3,” and “Brazil No. 5”; “Cuba No. 2-1” and “Cuba No. 2-2”; “Cuba No. 2-3,” “Havana-3,” and “YA2”; “Indonesia Bosuji” and “Indonesia No. 1”; “YA1,” “HONG HUA TIE GAN ZI,” and “H382”; “Segedinska Ruca” and “Scafati”; “YI SHUI DA WAN JIN” and “YI SHUI DA WAN JIN”; “PING GUO No. 21,” and “DAN ZHOU LIU DUI”; “GUI GANG No. 1” and “GUI GANG No. 2”; “BA SHU No. 1” and NY-3; “GU YIN No. 3,” “GU YIN No. 4,” “GU YIN No. 5,” “Cuba No. 8,” and “Cuba No. 9”; “XXHJH,” “Dominica Long Core Leaf,” “Nicaragua Short Core Leaf,” and “DAN ZHOU GUANG CUN”; “Criollo,” “SHAN DONG-1,” “SHAN DONG-2,” and “SHAN DONG-6”; “Havana No. 1,” “GBX1,” and “GBX2”; “Geudetthelmex,” “Havana10,” and “Lanka 23”; “Havana 211,” “Havana 38,” “Philippin,” and “Wisconsin 38”; “Mont Calme Brun,” and “XIN JING SI BIAO”; “Connecticut Shade” and “Bad Geudertheimer Landsorte”; “Havana IIc” and “Havana IIc”; and “Slovenia No. 1,” “SHI YAN No. 1,” and “LU YAN No. 1.” This means that the accessions in each group are suspected of being the same variety. Twenty-four pairs of candidate core primers were used to re-genotype the suspect varieties and their genetic distances were calculated (Supplementary Table 8). The results show that the genetic distances between “Brazil No. 2” and “Brazil No. 5”; “YA1,” “HONG HUA TIE GAN ZI,” and “H382”; “GUI GANG No. 1,” and “GUI GANG No. 2”; “YI SHUI DA WAN JIN,” and “YI SHUI DA WAN JIN”; “Cuba No. 2-1,” and “Cuba No. 2-2”; “GU YIN No. 3,” “GU YIN No. 4,” “GU YIN No. 5,” and “Cuba No. 9”; “Dominica Long Core Leaf,” and “Nicaragua Short Core Leaf”; “XXHJH” and “DAN ZHOU GUANG CUN”; “Criollo,” “SHAN DONG-1,” and “SHAN DONG-6”; “Havana No. 1” and “GBX1”; “BA SHU No. 1” and “NY-3”; “Connecticut Shade” and “Bad Geudertheimer Landsorte”; “SHI YAN No. 1,” and “SHI YAN No. 1”; “Geudetthelmex,” “Havana10,” and “Lanka 23”; and “Havana 38” and “Philippin 15” remained 0. These accessions were all grown in the field and further assessed, and combined with the field phenotypic traits, we found that the various index data for these varieties showed very few differences and they were difficult to distinguish, so the varieties in each group were determined to be the same variety.

## Discussion

### SNP-Based Genetic Relationships Among the Cigar Tobacco Accessions

Cigar tobacco germplasm resources in China are relatively scarce. Some of the varieties have been selected from local high-quality air-cured tobacco, but most of the others represent elite germplasm introduced from abroad. These cigar tobacco accessions provide a rich source of variation for cigar germplasm breeding. In order to facilitate the subsequent breeding and application of this germplasm, it is necessary to understand the genetic relationships and population genetic structure between varieties/accessions at the genomic level.

Using the nucleotide sequences of the variant site SNPs obtained by GBS for the 113 cigar tobacco accessions in this experiment as the sample sequences, we found that the results of PCA, genetic population analysis, and phylogenetic analysis are basically consistent and complement one another. The clustering results show that the 113 cigar accessions have no obvious correlation with geographical origin, indicating that the genetic background of these cigar tobacco accessions is complex and diverse and that cigar tobacco accessions originating from the same geographical regions are both related to each other and also to accessions from other independent groups. This result may be related to the genetic history of cigar tobacco. The main cultivated varieties in many countries were introduced directly from Cuba, Indonesia, the United States, and other countries or selected from varieties obtained from these countries. In the long-term selection process, cigar tobacco germplasm from different countries was introduced or exchanged. This germplasm exchange led to an insignificant correlation between the results of population genetic analysis and geographical origin. The results of our study provide a favorable reference and basis for better utilization of cigar tobacco germplasm resources in China, and this will be of great significance in the selection of parental lines in future cigar breeding programs and for the effective utilization of heterosis in the creation of new cigar tobacco varieties.

### Construction of SNP Fingerprints for the Cigar Tobacco Accessions

DNA fingerprints are based on nucleotide polymorphisms at multiple molecular marker loci distributed across the genome. The fingerprint map for cigar tobacco accessions is rich in polymorphism, has a high degree of individual specificity and environmental stability, and can discriminate between different individuals similar to a human fingerprint. Thus, molecular genotyping is referred to as “DNA fingerprinting” (Nybom et al., 2014). DNA fingerprinting has the advantages of being fast and accurate. In addition, DNA fingerprinting is a powerful tool for identifying varieties and strains, and it is also well suited for the identification of plant germplasm resources (Sorkheh et al., 2007; Gramazio et al., 2017; He et al., 2020; Wu et al., 2020). SNP marker-based high-throughput detection technology and data analysis is mainly based on the genetic characteristics of its “binary variation”—using two different fluorophores to label two different allelic variants—and a fluorescence detection system that can effectively distinguish two homozygous and one heterozygous genotypes. In order to meet the needs of large-scale detection and fingerprint database construction, a large number of SNP markers need to be developed and screened to achieve an ideal variety identification ability.

The choice of core fingerprinting markers depends on the complexity of the species genome, the marker type, the marker detection technology, and the number of varieties. In this study, KASP technology was used for SNP detection, and 47 core SNP sites were used to construct a fingerprint map of 216 cigar germplasm resources and to generate a unique two-dimensional barcode for each variety. These 47 KASP markers are highly polymorphic and have strong identification ability and can be directly used for variety genotyping and variety identification. At present, the National Tobacco Germplasm Bank and the Haikou Cigar Research Institute have preserved more than 300 cigar tobacco accessions. The Yunnan Provincial Tobacco Agriculture Research Institute and the Sichuan Deyang Tobacco Company have also preserved more than 140 and 100 cigar tobacco accessions, respectively. Therefore, methods to classify the cigar germplasm in these gene banks and to ensure the authenticity and purity of the seeds are the main concerns of cigar tobacco researchers and breeders. The two-dimensional barcode based on 47 core KASP markers and 216 cigar tobacco germplasm accessions may be very useful in the above work. However, there is still room for improving the SNP fingerprints. With the advances in breeding technology and the increase in the number of bred varieties, it may also be necessary to increase the number of SNP markers, especially those closely related to important agronomic traits, in order to identify the varieties more accurately and efficiently.

### Identification of 216 Cigar Tobacco Germplasm Accessions

Identification and screening of new varieties or cultivars is important in crop breeding; traditionally, varieties have been identified using morphological characters. When the plants grow to maturity in the field, after the characters are fully expressed, a large number of morphological characters can be used to identify different varieties. This method has the following shortcomings: first, the identification cycle is long and the cost is high; second, most of the traits investigated using morphological methods are quantitative traits that are controlled by multiple minor-effect genes and are therefore easily affected by the environment; third, in modern breeding programs, new varieties are mostly selected from crosses involving a limited number of elite parental lines. The genetic variation present in new varieties is decreasing, and the morphological differences between varieties that can be used for variety identification are smaller and fewer. The lack of distinct morphological traits increases the difficulty of variety identification. Through the application of biotechnology, a series of DNA molecular marker technologies have been developed that have brought great changes to crop genetics and breeding research and have also been applied to variety identification. Among these marker systems, SNPs have the advantages of large numbers, wide genomic distribution, high stability and heritability, and straightforward identification for use in rapid and high-throughput genotyping assays (Rafaiski, 2002; Batley and Edwards, 2007; Hayward et al., 2012). SNP markers are based on single-nucleotide mutations that have a low mutation frequency and high genetic stability. SNPs have shown great potential when applied to variety identification and can be used to supplement traditional variety identification methods (Lee et al., 2008; Huang et al., 2009; McCouch et al., 2010; Fernandez i Marti et al., 2012; Poland and Rife, 2012; Subbaiyan et al., 2012; De Donato et al., 2013; Alipour et al., 2017; Zhang et al., 2020).

In China, cigar tobacco can only be identified using morphological characters at present, which are greatly affected by external environmental conditions and cultivation practices and are prone to scoring errors which can lead to inaccurate results. To date, no SNP-based barcoding system has been implemented for cigar tobacco varieties. Here, for the first time, we screened a set of SNP markers to develop a SNP-based barcode for cigar tobacco identification. The 47-SNP barcode is rich in variations and has high power to discriminate the 216 cigar tobacco accessions from one another. On average, one SNP locus can identify five accessions, a resolution capacity that is much higher than that reported for ISSRs (Yang et al., 2010) and SSRs (He et al., 2020), which have been used for the identification and evaluation of tobacco accessions in the past. A SNP barcode provides a tool to discriminate very closely related accessions or the origins of the cigar tobacco subgenomes. In the present study, the cigar tobacco accessions used for generating the SNP-based barcodes represent collections of the main varieties from several high-quality cigar-producing areas of the world. These accessions show considerable variation and include both landraces and introduced cultivars. The 47-SNP-based barcodes can discriminate closely related varieties from one another, such as “Cuba No. 5” and “Cuba No. 6.”

Variety identification should not only be accurate, reliable, and fast but also be simple, and the technology should lend itself to automation, which is the trend in variety identification technology. Therefore, SNP markers can be used to identify the existing cigar tobacco germplasm resources in China, to screen out duplicate germplasm, and to establish a foundation for the standardization of China's cigar tobacco germplasm resources and their use in subsequent molecular genetic breeding programs. At present, high-throughput detection of SNP markers in large sample sets has shown advantages in rapid variety identification. In the future, as the number of varieties increases and more SNP markers are developed, high-throughput SNP detection technology will have a very broad application in the fingerprinting and identification of newly developed cigar tobacco varieties.

## Conclusion

Our study has demonstrated that GBS is a powerful tool for investigating population structure and genetic diversity in cigar tobacco cultivars. In this study, 113 cigar tobacco accession collected from major domestic breeding units were divided into four well-separated clusters, and we found no significant correlation between the group distribution and the geographical origin of the cigar germplasm. We obtained 47 core KASP markers and 24 candidate core markers and constructed SNP fingerprints of 216 cigar germplasm resources. Our study is a valuable addition to the present genomic resources of cigar tobacco, and these molecular markers are tools for cultivar identification, determining genetic diversity and relatedness among cultivars, and for testing the authenticity and purity of cigar tobacco inbred and hybrid lines.

## Data Availability Statement

The datasets presented in this study can be found in online repositories. The names of the repository/repositories and accession number(s) can be found below: NCBI SRA, PRJNA673075.

## Author Contributions

GL and XZ conceived the experiments and secured the funding. YW performed the experiments. YW and HL analyzed the data and drafted the manuscript. XX, AY, QF, PD, YL, and XJ contributed to conceive the project and revise the paper. All authors contributed to the article and approved the submitted version.

## Conflict of Interest

The authors declare that the research was conducted in the absence of any commercial or financial relationships that could be construed as a potential conflict of interest.

## Supplementary Material

Supplementary Table 1 113 cigar tobacco germplasm resources for GBS sequencing.

Supplementary Table 2 Core KASP primer validation group.

Supplementary Table 3 216 cigar germplasm resources used to construct fingerprints.

Supplementary Table 4 Sequencing data of 113 test materials.

Supplementary Table 5 47 pairs of core KASP primers.

Supplementary Table 6 24 pairs of candidate core KASP primers.

Supplementary Table 7 Genetic distance of 216 cigar germplasm resources based on 47 core KASP primers.

Supplementary Table 8 The genetic distance between 56 suspected varieties based on 24 candidate KASP primers.

Supplementary File 1 2D barcodes for 216 of the cigar tobacco accessions.
