# Supplementary material for: Construction of a SNP Fingerprinting Database and Population Genetic Analysis of Cigar Tobacco Germplasm Resources in China
Source: Front Plant Sci. 2021 Feb 24;12:618133. doi: 10.3389/fpls.2021.618133 (PMC7943628; doi:10.3389/fpls.2021.618133)
Supplement: Supplementary Table 1 — 113 cigar tobacco germplasm resources for GBS sequencing. [file Data_Sheet_1.ZIP › File S1.pdf]

B001	B002	B003	B004
B005	B006	B007	B008
B009	B010	B011	B012
B013	B014	B015	B016
B017	B018	B019	B020

B021 B022 B023 B024

B025 B026 B027 B028

B029 B030 B031 B032

B033 B034 B035 B036

B037 B038 B039 B040

B041 B042 B043 B044

B045 B046 B047 B048

B049 B053 B054 B055

B056 B057 B058 B059

B060 B061 B062 B063

B064 B065 B066 B067

B068 B069 B070 B071

B072 B073 B074 B075

B076 B077 B078 B079

B080 B081 B082 B083

B084	B085	B086	B087
B088	B089	B090	B091
B092	B093	B094	B095
B096	B097	B098	B099
B100	B101	B102	B103

B104 B105 B106 B107

B108 B109 B110 B111

B112 B113 B114 B115

B116 B118 B119 B120

B121 B122 B123 B124

B125 B127 B128 B129

B131 B132 B133 B134

B135 B136 B137 B138

B139 B140 B141 B142

B143 B144 B145 B146

B147 B148 B149 B150

B151 B152 B153 B154

B155 B156 B157 B158

B159 B160 B161 B162

B163 B164 B168 B169

B170	B171	B172	B173
B174	B175	B176	B177
B178	B179	B180	B181
B182	B183	B184	B185
B186	B187	B188	B189

B190	B191	B192	B193
B194	B195	B196	B197
B198	B199	B200	B201
B202	B203	B204	B205
B206	B207	B208	B209

B210	B216	B217	B229
B230	B231	B232	B233
B234	B235	B236	B237
B251	B252	B255	B257
